# Effect of Pollen Limitation and Pollinator Visitation on Pollination Success of *Haloxylon ammodendron* (C. A. Mey.) Bunge in Fragmented Habitats

**DOI:** 10.3389/fpls.2019.00327

**Published:** 2019-03-22

**Authors:** Min Chen, Xiao-An Zuo

**Affiliations:** Cold and Arid Regions Environmental and Engineering Research Institute, Chinese Academy of Sciences, Lanzhou, China

**Keywords:** habitat fragmentation, pollen limitation, pollinator, pollinator visitation rate, seed set

## Abstract

*Haloxylon ammodendron* (C. A. Mey.) Bunge is an ecologically important species in arid regions. Pollen limitation may decrease plant reproduction due to low levels of pollen transfer and inadequate pollen receipt. In arid regions, pollen limitations of many plant species may be influenced by habitat fragmentation. However, whether pollen limitation and pollinator visitation affect the pollination success of *H. ammodendron* (Amaranthaceae) in fragmented habitats still needs further study. In this study, we calculated the pollen limitation in natural and fragmented habitats to estimate the effect of habitat fragmentation on pollen limitation. In different habitats, we investigated the relationship between the number of open flowers and pollinator visiting frequency. In addition, we examined how habitat fragmentation affects pollination success through the influence of pollinator visitation rate on seed set. Our results indicated that pollen limitation was the important limiting factor for seed set in fragmented and natural habitats. The results showed higher pollinator visitation rates resulted in a higher percentage of seeds in both habitats. In *H. ammodendron*, *Apis mellifera* was found to be the dominant pollinator. These results may support the assertion that plants evolve traits to attract pollinators and pollinators increase their visiting frequency to better exploit the floral resources. We also determined that outcrossing was dominant in the breeding system and that wind pollination played an important role in pollination success. This study aims to contribute to a better understanding of how environmental heterogeneity affects pollen limitation, pollinator visitation, and pollination success in arid regions.

## Introduction

In many flowering plants, a large proportion of flowers do not develop into fruits and seeds (Stephenson, 1981; Larson and Barrett, 2000). Many hypotheses have been presented to explain this phenomenon, and one prominent hypothesis is that pollen limitation may result in low fruit and seed set (Burd, 1995; Chen et al., 2015). Over the past decade, there has been a substantial increase in the research investigating the effect of pollen limitation on reproductive success (Ashman et al., 2004; Gómez et al., 2010). Pollen limitation has received special attention because plants rely on abiotic or biotic vectors to transport pollen for sexual reproduction, and inadequate pollen quality and/or quantity can lead to low reproductive output (Knight et al., 2005; Gómez et al., 2010). Many plants under natural pollination conditions suffer from pollen limitation, especially when their habitats change (Eriksson and Jakobsson, 1998; Hill et al., 2008).

Many studies have indicated that floral traits may affect not only pollinator activity and visitation but also pollination efficiency (Muchhala, 2007; Sletvold et al., 2010). Ferdy et al. (1998) and Biernaskie et al. (2009) also noted that many flowers are avoided after a few visits because most pollinators have strong associative learning abilities, and most insect-pollinated plants show evidence of inadequate pollen receipt. Pollen limitation has also been widely observed and often interpreted as evidence for insufficient pollinator visitation in fragmented habitats (Aguilar et al., 2006; Nayak and Davidar, 2010; Wagenius and Lyon, 2010). Human impacts on habitats usually result in the fragmentation and isolation of ecosystems (Saunders et al., 1991). Moreover, fragmented habitats can change the foraging patterns of pollinators and affect pollinator behavior (Cresswell, 1997; Rodríguez-Cabal et al., 2007). Kormann et al. (2016) suggested that the breakdown of pollination mutualisms because of habitat loss and fragmentation results in reduced pollinator density and visitation frequency. Habitat changes that increase or decrease plant density may subsequently influence the availability of pollinators and pollination success, and may even trigger the local extinction of plants in arid regions (Hadley and Betts, 2012).

*Haloxylon ammodendron* (Amaranthaceae) has great potential for livestock feed and medicinal use, and it plays an important role in sand fixation and vegetation productivity because its root system is very efficient at absorbing water, which makes it both drought- and salt-resistant in arid regions (Song et al., 2012). *H. ammodendron* is a species of Amaranthaceae. In Amaranthaceae, a large number of plants are gynodioecious species and some dioecious. Dufaÿ et al. (2008) have suggested that gynodioecy is a plant breeding system where females and hermaphrodites coexist in populations. In the reproductive history of angiosperms, the evolution from hermaphroditism to dioecy (separate sexes) is considered one of the most important evolutionary transitions (Dufaÿ et al., 2008). Dufaÿ et al. (2014) also indicated that the gynodioecy pathway is not restricted to a few taxa but may instead be widespread in angiosperms.

This study aimed to investigate the effect of pollen limitation and pollinator visitation on the seed set of *H. ammodendron* in a fragmented habitat. Our specific objectives were to (1) determine the possible differences in pollen limitations between two different habitats and whether resource reallocation can alter seed set per flower under pollen supplementation; (2) examine the relationship between the mean monthly rainfall and proportion of flowers in anthesis; (3) analyze how habitat fragmentation affects the number of flowers and pollinator visitation; and (4) estimate how pollinator visitation rate influences seed set per flower. Furthermore, we discussed pollinators and the pollination success of *H. ammodendron* in a natural habitat and a fragmented habitat.

## Materials and Methods

### Study Site and Fragmentation Experiment

The study site is in the grasslands of the Urat Desert in western Inner Mongolia, China (41°06′–41°25′N, 106°59′–107°05′E). The annual mean potential evapotranspiration is approximately 2,200 mm, and the annual mean rainfall is approximately 153.9 mm.

**Figure 1:**
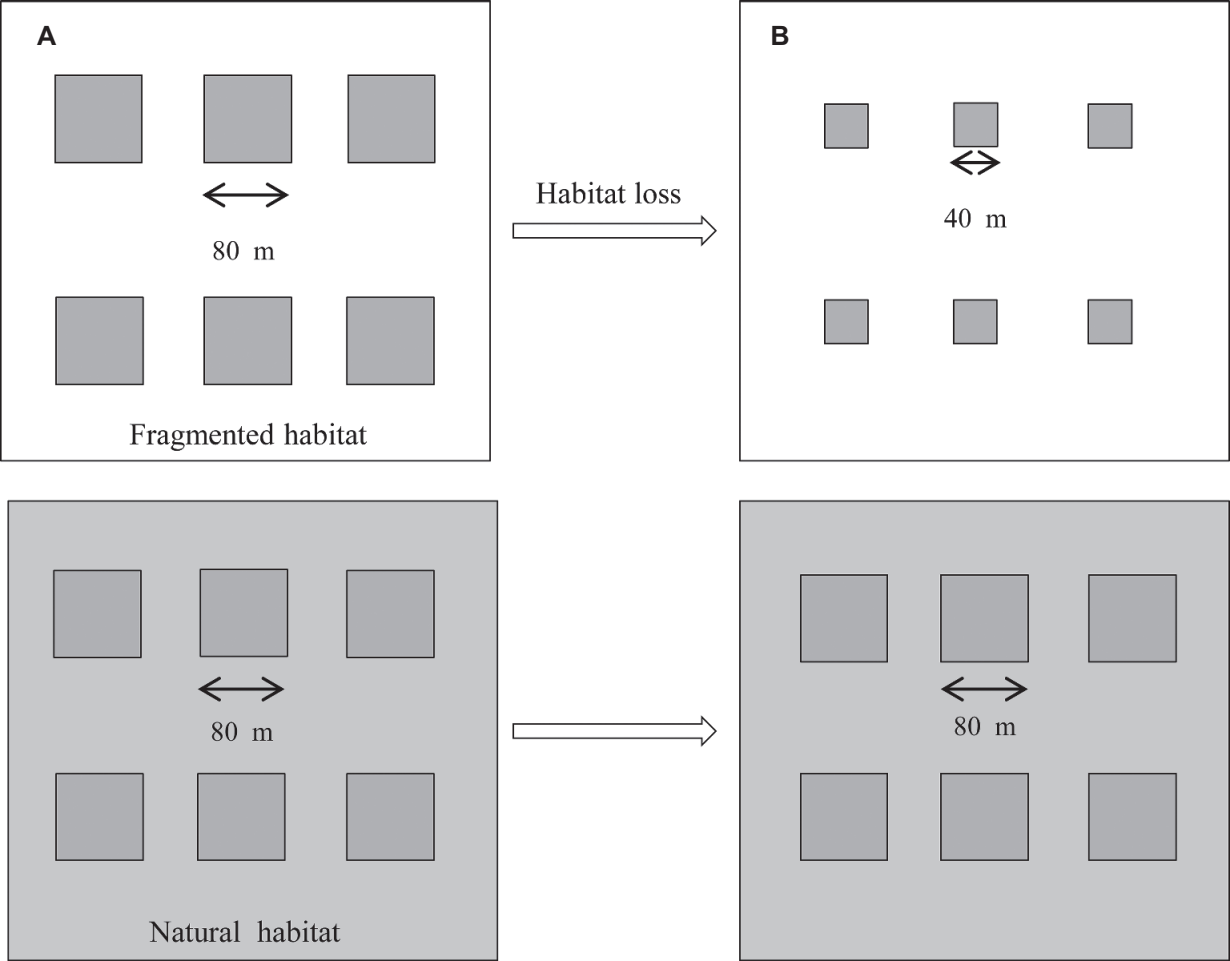
Experimental layout for the two studied patches, the fragmented and natural patches, from 2014 to 2017. The six fragmented plots (80 m × 80 m) were separated by mown vegetation (white area). The control plots were symmetrically arranged and surrounded by undisturbed vegetation (gray area). **(A)** Plot size of the studied habitat (80 m × 80 m) in 2014. **(B)** Plot size of the studied habitat (40 m × 40 m) in 2017.

This study was carried out from April 2014 to October 2017. The original design consisted of two studied habitats containing a total of 12 plots in the nutrient-poor, dry grasslands. The six plots in the fragmented habitat were divided by areas of vegetation that were frequently mown (five times per year). The corresponding natural plots were symmetrically arranged and surrounded by undisturbed vegetation and had the same plant community as the fragmented habitat (Figure 1), but it was bordered by undisturbed vegetation. The average number of *H. ammodendron* plants was similar among the studied plots, and the distance between the plots was approximately 60–100 m. In each plot, the average density of *H. ammodendron* was 15 individuals per 100 m^2^ in 2014. In 2017, we observed the average density of *H. ammodendron* was 14 individuals per 100 m^2^ in fragmented plots. In addition, the two studied habitats were separated by 800 m. From 2014 to 2017, the fragmented habitats were disrupted by a decrease in plot size from 6,400 to 1,600 m^2^ due to the vegetation reduction in the area. In *H. ammodendron*, flowers in anthesis typically occur from May until July, and the fruiting period is from September to October.

### Traits of Floral and Daily Flowering Period

*H. ammodendron* has yellowish, bisexual flowers with five stamens and one pistil. Ten labeled plants per plot were collected and observed for daily flowering period characteristics. Flowers in the anthesis were counted throughout the entire reproductive season (from April to October) for each plant on each plot. We calculated the mean monthly rainfall and the number of open flowers per inflorescence in the natural and fragmented habitats. In order to observe the number of open flowers per inflorescence in both habitats, we labeled three flowering plants (three inflorescences per plant) in each plot. The floral observations were made over 2 weeks, and data regarding the phases of flowering, the number of open flowers (*n* = 108 inflorescences), and the flowering time were recorded. These measures have been carried out in each year (from 2014 to 2017).

To assess floral traits in the daily flowering period and anther dehiscence, six budding plants were randomly labeled in each plot. In total, we selected 72 plants and measured three fully developed flowers from each of the plants (*n* = 216 flowers). Floral phenology (anthesis and anther dehiscence) was studied on the marked flowers and observed from 06:00 to 19:00. To evaluate the daily flowering period, these measured flowers were recorded over 12 days after flower opening. We conducted daily inspections following 216 tagged flowers growing in each month (2 weeks per month) until wilting during October.

### Quantifying Pollen Limitation

To determine the effects of habitat types (natural and fragmented) and treatments (pollen added, control, and procedural control) on seed set in each year (from 2014 to 2017), we conducted the pollen supplementation experiment during the flowering days. For the addition of pollen, we harvested fresh pollen from plants growing at least 10 m away from the experimental plants until sufficient pollen grains were on the surface of the stigma. To detect the possible effect of resource allocation, we used two complementary controls: one from the manipulated plants as a control and the other from non-manipulated plants as a procedural control (Ashman et al., 2004; Wesselingh, 2007).

In each plot, we marked one branch (with eight marked flowers) of each of the eight labeled plants at the same flowering stage. In four of the eight labeled plants, we marked eight flowers from the central part of the flowering stalk, adding outcrossed pollen to the lower four flowers as the pollen-added treatment (PA treatment) and leaving the upper four flowers as the control (C treatment). In the four remaining labeled plants, the upper four flowers on each plant acted as the procedural control (CC treatment) and the lower four flowers are natural condition. We selected 96 branches (two habitats, six plots per habitat, eight plants per plot, one branch per plant), and measured 12 labeled flowers (four flowers per treatment) from two branches. In total, we measured 576 flowers in each year.

To determine whether pollen limitation affected reproductive success, we investigated the mean seed set of these treatments in each year (from 2014 to 2017). Pollen limitation was estimated based on seed set according to Larson and Barrett (2000):

$${\mathrm{PL}}_{C}=1-\left({\mathrm{RS}}_{C}/{\mathrm{RS}}_{\mathrm{PA}}\right)$$

where RS_C_ is the seed set under the control treatment and RS_PA_ is the seed set under the pollen-added treatment. Positive values indicate a higher reproductive success in the PA treatment than in the C treatment and therefore pollen limitation. By contrast, a value of zero or negative values indicate there is no pollen limitation.

### Observing Pollinator Activity

From May to July, we randomly selected three plants in each plot to determine the identity and surveys of pollinators. We performed 12 h focal observations, at different periods of the day, from 6:00 to 10:00 (morning), 10:00 to 14:00 (noon), and 14:00 to 18:00 h (afternoon) on different days. We used fixed video cameras to record the duration of each pollinator visit, time until some of them visited the plot, and the number of plants and number of flowers visited per foraging bout. In each habitat, six surveyors used 168 h (12 h per day) to record pollinator activity because each observation period was 2 weeks in each month. Pollinators were captured using insect nets for later identification in the laboratory. We carefully analyzed the presence or absence of pollen grains adhering to the bodies of the pollinators and determined whether they contacted stamens and stigmas. Pollen was collected by using a cube of fuchsin-stained jelly to rub the insect body following the Beattie’s method (Beattie, 1971). Assigned observers counted the quantity and activity of the pollinators, and we used DAT recorders to measure pollinator visit durations. The visiting frequency of the pollinators (*V*_f_) was calculated according to the following equation (Goverde et al., 2002):

$$\mathrm{Visiting frequency}=\frac{\;\mathrm{Number of visits}\;}{\;\mathrm{Number of flowers}\times \mathrm{Observation time}\;}$$

### Effect of Pollinator Visitation on Seed Set

To determine how pollinator visitation affected seed set in each year (from 2014 to 2017), we marked six plants in the natural habitat and six plants in the fragmented habitat while they were still flowering. For each plant, 10 flowers were randomly chosen and marked with tags. We noted the flowering stage and growth progress of the marked flowers (*n* = 120 flowers) based on recorded films. Moreover, we investigated the proportions of open flowers and the pollinator visitation and seed production from May to September, when all seeds were mature. The fruits were collected in early October, and the length of the fruits (utricle) and the number of seeds were examined in the laboratory. Ovary enlargement was used as a valid criterion for assessing the fertilization of ovules (Suzuki, 2000). In the bagged treatment, the flower buds were covered with bags to prevent insect visits and wind pollination. We measured the length of the ovaries in the bagged and non-pollinated flowers when the other flowers were fruiting. The average length of the ovaries was 2.5 ± 0.31 mm (*n* = 20, mean ± SE). Flowers with ovaries (utricle) >3 mm were used as a range for successfully pollinated. The percent of fresh flowers and mature seeds were recorded and calculated according to the following equation (Suzuki, 2000):

$$\mathrm{Percentage of seeds among visited flowers}=\frac{S}{\;V\;}\times 100\%$$

where *V* is the proportion of visited flowers and *S* is the proportion of seeding flowers on marked plants.

### Hand Pollination Experiments

We determined the pollination success of *H. ammodendron* by monitoring the seed set in different pollination experiments. In each plot, one marked branch (with three marked flowers) on each of the six labeled plants was identified before anthesis in each year (from 2014 to 2017). On the same plant in each plot, one marked branch (*n* = 3 flowers) was used for natural pollination; one branch (*n* = 3 flowers) was used for wind pollination, for which the stamens were removed prior to the release of pollen; and the flowers were covered in a mesh bag (1 mm^2^) to prevent visits by insects (Krishnana et al., 2012).

In each plot, we selected two branches (*n* = 3 flowers per branch) per labeled plant, with one branch for the cross-pollination treatment and the other branch for the self-pollination treatments. Moreover, we used fresh flowers as the pollen source for artificial pollination, with flowers collected from different plants for the cross-pollination treatment and from the same plant for the self-pollination treatment. In cross-pollination treatment, where the stigmas of the emasculated flowers were hand-pollinated using pollen obtained from different flowers, the flowers were bagged, and three opening flowers of the same inflorescence were considered different flowers in instances when anthesis had not begun in most flowers in order to ensure the pollen supplementation success of recipient flowers. In the natural pollination and self-pollination treatments, the branches were covered with paper bags to exclude all pollinating agents. We selected 144 branches (two habitats, six plots per habitat, three plants per plot, one branch per treatment, and three flowers per branch). In total, we measured 432 flowers in each year. In September, we counted all seeds from the treatments in the laboratory.

### Data Analyses

A general linear model was used to and to compare the number of open flowers per inflorescence between the natural and fragmented habitats in each year (from 2014 to 2017). The model used the habitat types and years as fixed factors, and it used the number of open flowers per inflorescence as the dependent variable.

We used a linear mixed model analysis of variance and added a random effect (No. tagged) describing the focal plant effect. No. tagged was used as a random factor among the two habitats. The model was performed with gamma distribution and a logit link function. Then, we used GLM model to assess differences in seed set of each observed flower. The model used the habitat types (natural and fragmented), years (from 2014 to 2017), and treatments (PA, C, and CC) as fixed factors, and it used the seed set as the dependent variable. We used likelihood ratio test in the model, and differences between treatments were evaluated with Tukey multiple comparisons.

A GLM model was performed with binomial error and a logit link function. The GLM model included habitat types and years treated as fixed effects, and number of flower visited as a binary response variable. We used likelihood ratio test in the model. In addition, a Tukey’s *post hoc* test was used for multiple comparisons among pairs of means of flower visited.

To assess differences in seed set among pollination treatments, a GLM was performed with gamma distribution and a logit link function. The GLM model included pollination treatment, habitat types (natural and fragmented), and years (from 2014 to 2017) treated as fixed effects, and it used the seed set as the dependent variable. We used likelihood ratio test in the model. A Tukey *post hoc* test was used for multiple comparisons among pairs of seed set of pollination treatments. All analyses were performed by using the SPSS 19.0 statistical software package.

## Results

### Phenology and Floral Traits

**Figure 2:**
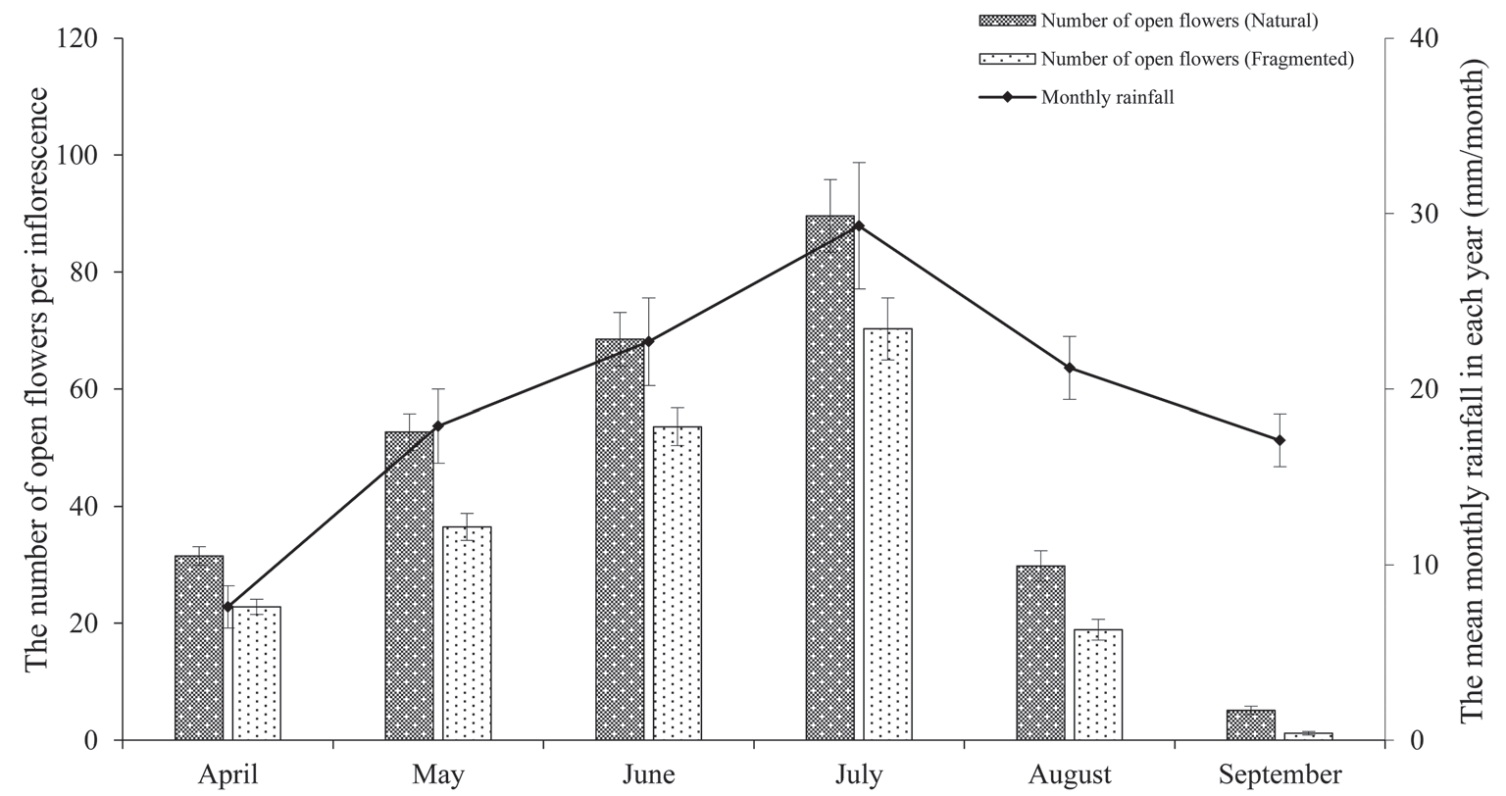
Relationship between the mean monthly rainfall and the number of open flowers per inflorescence in the studied habitats. The monthly rainfall and the number of open flowers per inflorescence are the means of data from 2014 to 2017.

In the studied habitats, the flowering of *H. ammodendron* typically occurs from mid-June until late July, and the period of peak flowering was found to coincide with the monthly rainfall (Figure 2). We observed that the number of open flowers per inflorescence in the natural and fragmented habitats was (mean number ± *SD*) 46.2 ± 9.3 and 33.9 ± 5.7, respectively. We found significant differences in the number of open flowers per inflorescence between the natural and fragmented habitats (GLM, habitat types effect, *df* = 1, *F* = 114.582, *p* < 0.001; Table 1).

**Table 1:** Effect of habitat types (natural and fragmented) and years (from 2014 to 2017) on the number of open flower per inflorescence in *H. ammodendron*.

	Number of open flowers			
	*df*	MS	F	*p*
Habitat types (H)	1	908.970	114.582	<0.001
Years (Y)	3	357.257	45.035	<0.001
H × Y	3	36.710	4.628	0.016

In the studied habitat, we observed a daily flowering period of 528 individual flowers. For most flowers, it took approximately 7 days from corolla opening to stamen and petal wilting. We observed that most *H. ammodendron* flowers had a diurnal anthesis, and the corollas began to open at approximately 08:00 and were completely open at approximately 09:00. In addition, we found that corollas began to close at approximately 15:00 and were completely closed at approximately 18:00. After 3 days, the anthers and stigma were completely dry.

### Pollen Limitation

**Figure 3:**
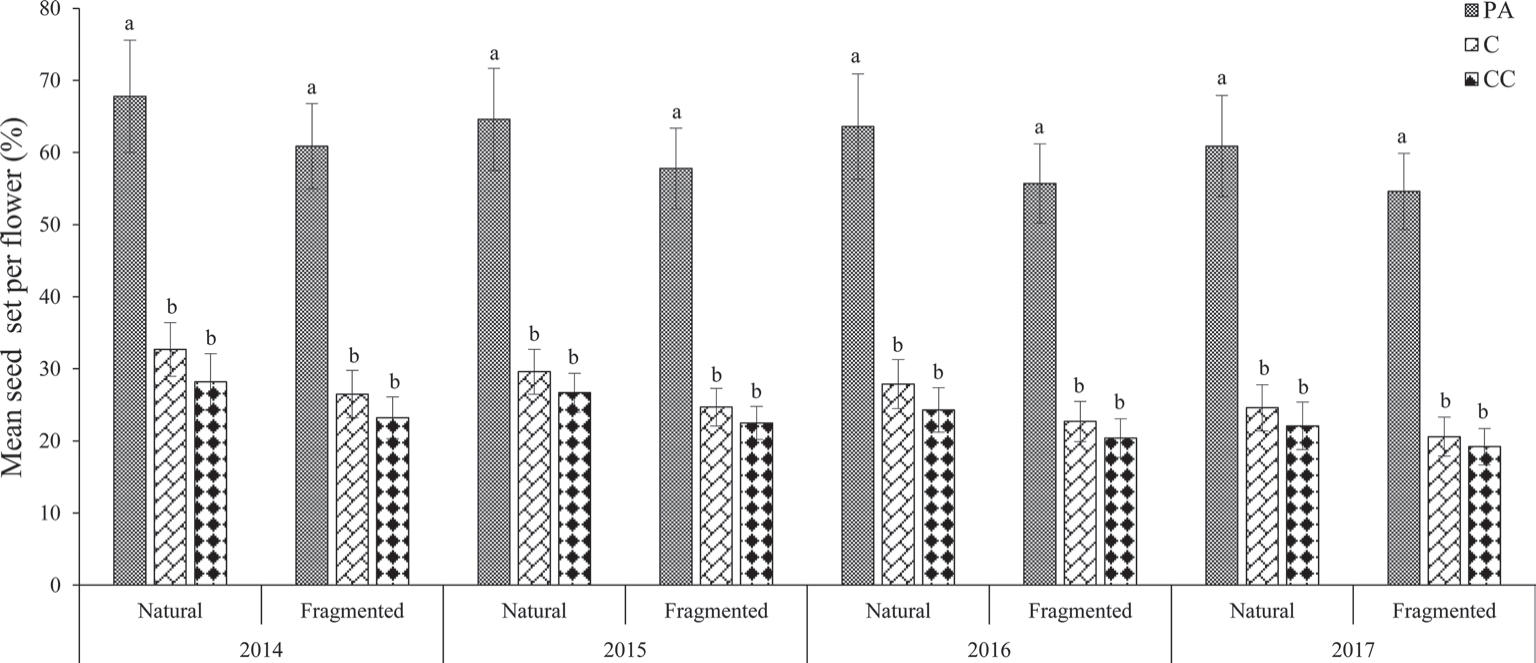
Mean seed set of *H. ammodendron* under the pollen limitation treatments in each year (from 2014 to 2017). Vertical bars denote the standard error. PA, pollen addition; C, control; CC, procedural control. Different letters show a significant difference at the 0.01 level.

In the natural habitat, the mean seed sets were 28.7 ± 3.5% (mean number ± *SD*) in the control and 64.2 ± 7.5% in the pollen addition treatment (Figure 3). In the fragmented habitat, the mean seed sets did differ significantly between the control and pollen addition treatment according to the GLM model, at 23.6 ± 2.9% (control) and 57.3 ± 5.2% (pollen added; GLM, treatments effect, likelihood ratio *χ^2^* = 107.221, *df* = 1, *p* < 0.001). In both habitats, pollen supplementation significantly increased the mean seed set between the control and pollen addition treatment from 2014 to 2017 (GLM, treatments effect, likelihood ratio *χ^2^* = 300.89, *df* = 2, *p* < 0.001; Table 2).

**Table 2:** Effect of habitat types (natural and fragmented), treatments (PA, C, and CC), and years (each year from 2014 to 2017) on the seed set of *H. ammodendron*.

	Seed set		
	Likelihood ratio χ^2^	*df*	*p*
Types	33.188	1	<0.001
Years	2.535	3	<0.001
Treatments	300.894	2	<0.001

PA, pollen added; C, control; CC, procedural control.

In the natural habitat, the mean seed set did not differ significantly between the control and the procedural control flowers, with values of 28.7 ± 3.5% for the control and 25.3 ± 3.2% for the procedural control (GLM, treatments effect, likelihood ratio χ^2^ = 2.72, *df* = 1, *p* > 0.05). In the fragmented habitat, the mean seed set was 23.6 ± 2.9% in the control and 21.3 ± 2.6% in the procedural control treatment. We found the control and the procedural control did not differ statistically in terms of seed-set. Our results indicated that the pollen limitation index was 0.553 ± 0.076 in the natural habitat and 0.588 ± 0.082 in the fragmented habitat. Based on these PL index values, pollen limitation was the important limiting factor for seed set, but pollen limitation was similar in the fragmented and natural habitats.

### Pollinator Visitation

**Figure 4:**
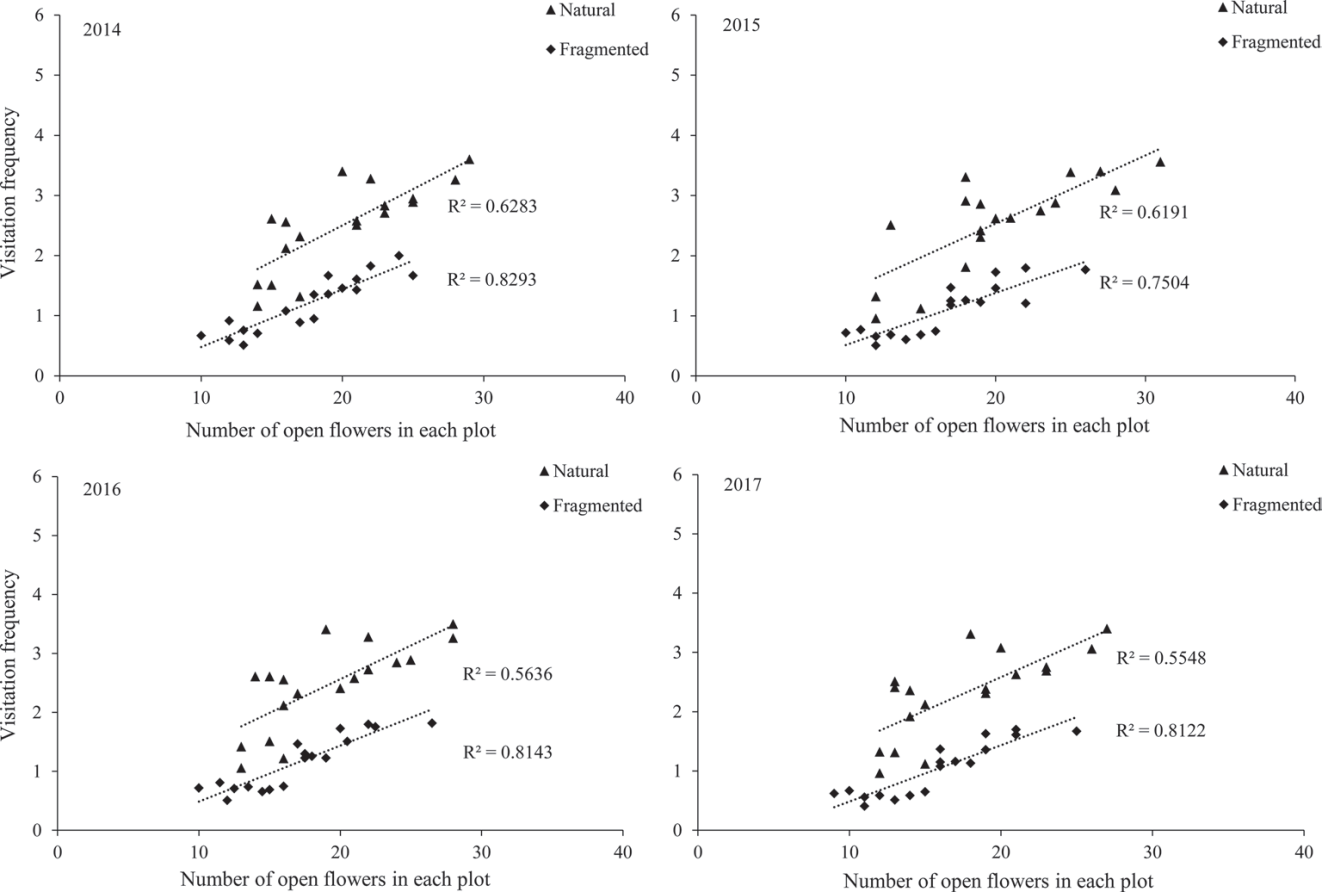
Relationship between visitation frequency and number of open flowers in the studied habitats from 2014 to 2017.

**Figure 5:**
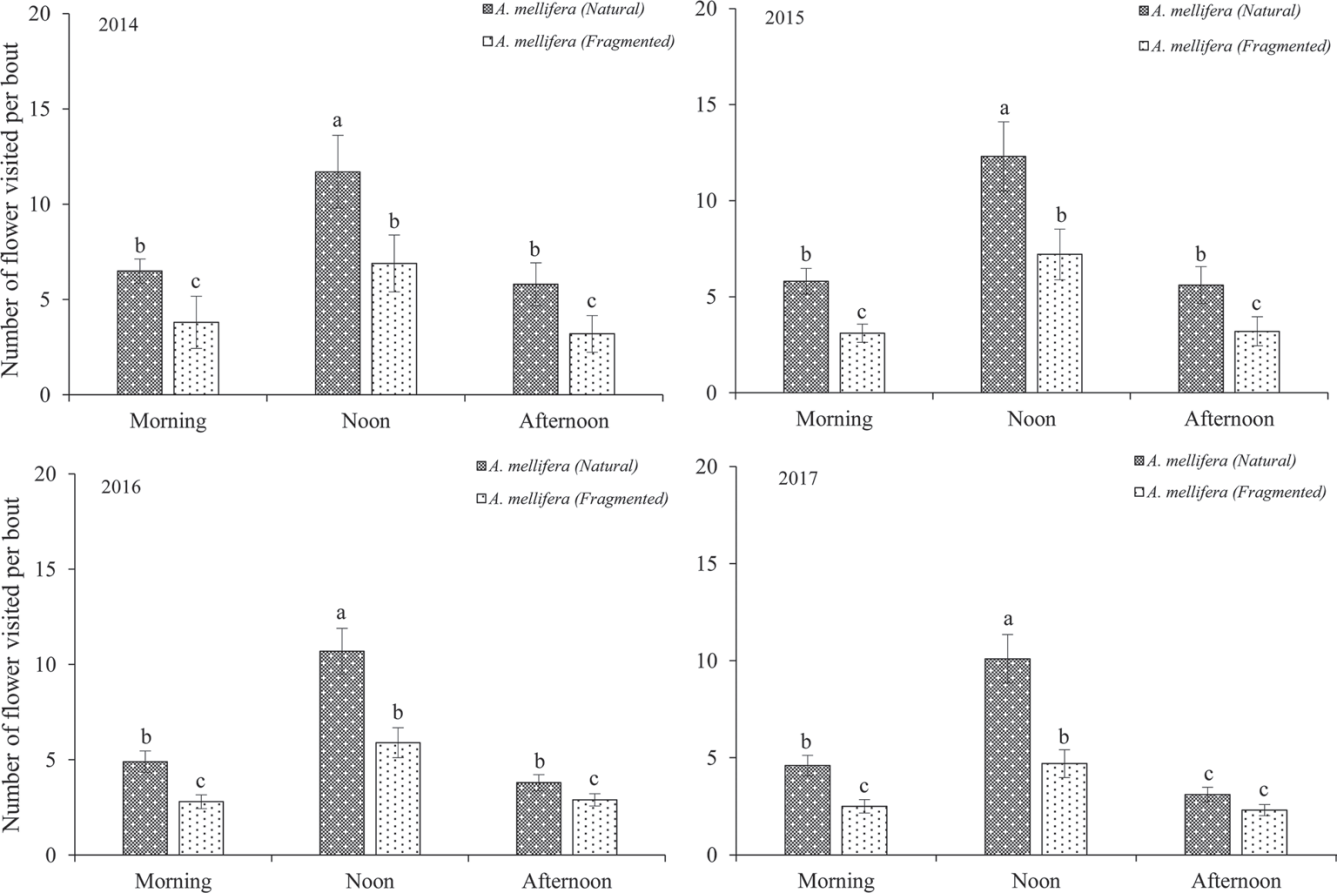
Frequency of dominant pollinator (*A. mellifera*) visits to *H. ammodendron* throughout the day. Number of flowers visited per bout. Values (mean ± *SD*) with the same superscript letters are not significantly different between groups ( *p* > 0.05).

Our results indicated that bees (Hymenoptera) accounted for 91.2% of all 216 floral visitors observed, 57.3% of the total visitors were *Apis mellifera* and 23.7% of the total visitors were *Megachile spissula* Cockerell. Other occasional visitors (10.2% of the total) included *Episyrphus balteatus* and *Pieris rapae* Linne (Supplementary Material), but these species only play an assistant role in pollination success due to their infrequent visitation and because they rarely touch the stigma or anthers. We found *A. mellifera* was the dominant pollinator and intensively visited flowers. In *A. mellifera*, the mean visiting frequency is 2.6 visits/(hour × flower), and other pollinators showed visiting frequencies lower than 1.2 visits/(hour × flower). Our results indicated that a positive relationship between the pollinator visiting frequency and the number of open flowers in the natural and fragmented habitats (Figure 4). The flower opening and pollen release occurred between 08:00 and 15:00, and the most frequent activity of *A. mellifera* coincided with this period in each year from 2014 to 2017 (Figure 5).

### Pollinator Visitation Rate Affects Seed Set

In the natural habitat, 38.56% of the mean flowers were visited (*V*) at least once by effective pollinators, and 20.35% of the mean flowers produced seeds (*S*), resulting in a mean seed set percentage for the visited flowers (*S*/*V* × 100%) of 52.77%. In the fragmented habitat, our results showed that 29.18% of the mean flowers were visited, 14.51% of the mean flowers produced seeds, and a mean seed set percentage for the visited flowers was 49.73%. These outcomes showed that higher pollinator visitation rates resulted in a higher percentage of seeds in both habitats.

### Breeding System

**Figure 6:**
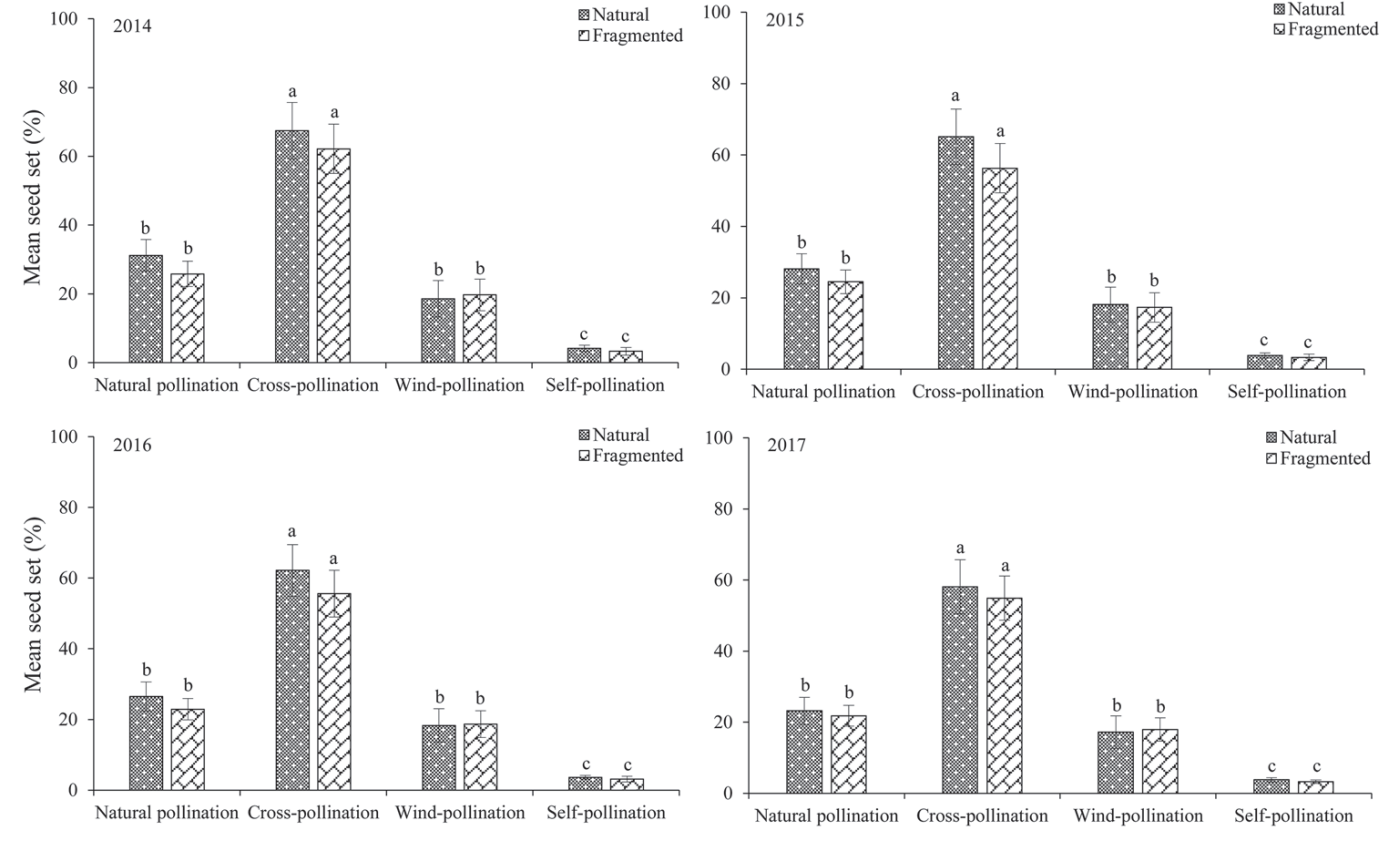
Seed set in *H. ammodendron* by pollination treatment from 2014 to 2017. Values (mean ± *SD*) with the same superscript letters are not significantly different between groups (*p* > 0.05).

The seed set obtained for each pollination treatment in each year (from 2014 to 2017) is shown in Figure 6. In the studied habitats, the seed sets were significantly higher in the cross-pollinated treatments than in the naturally pollinated treatments (GLM, pollination treatments effect, likelihood ratio *χ^2^* = 128.50, *df* = 1, *p* < 0.001), suggesting outcrossing successfully promoted pollination success. In addition, there was no significant difference in the seed set of natural pollination between natural and fragmented habitats (GLM, habitat types effect, likelihood ratio χ^2^ = 2.62, *df* = 1, *p* > 0.05).

In the natural habitat, natural and wind-pollinated flowers had mean seed set values of 27.3 ± 3.8 and 18.1 ± 4.9%, respectively. Moreover, the mean seed set of the self-pollinated flowers was only 3.9 ± 1.2% in the natural habitat. Our results indicated that anemophily suffices for pollination in this species.

## Discussion

### Floral Traits and Pollinators

In flowering plants, floral attraction affects pollinators and pollination efficiency, and floral traits are related to the environment and climate change (McCarty, 2001; Ashman and Morgan, 2004; Sinu and Shivanna, 2007). Melampy (1987) suggested that the flower bloom response to rainfall is considered to be an adaptive process, in which increased rainfall can increase the proportion of blooming flowers. We also found that the blooming flowers are adapted to increased monthly rainfall in arid environments. Ortíz et al. (2010) showed that differences in visiting frequency and pollinator behavior could be associated with the density of flower resources. More specifically, floral traits as well as the anthers attract flower visitors to specific flowers, and the targets of pollinators are nectar and pollen. In addition, habitat fragmentation could significantly affect the proportion of flowers in anthesis, and flowers in natural habitats have more floral resources than those in fragmented habitats. These study results may explain why there was a positive relationship between the pollinator visitation frequency and number of flowers in anthesis and why the number of flowers visited by *A. mellifera* in the natural habitat was significantly higher than that in the fragmented habitat.

A plant’s probability of being visited by effective pollen vectors increases with pollinator activity (Perfectti et al., 2009). Often, in plants, there is a convergent evolution of floral traits to match the traits of their common pollinators, one of the most visual testimonies to natural selection (Pauw et al., 2009). Our results indicated that pollination was more efficient when the pollinator visits were concentrated because the filaments in plants dry easily in arid regions. In this study, flowers completely opened, enabling pollen release, between 09:00 and 15:00, representing an important period for the pollination success of *H. ammodendron*. In addition, the time of *A. mellifera* activity coincided with this period.

### Pollen Limitation and the Reallocation of Resources Affect Plant Reproduction

A plant is considered pollen-limited if pollen supplementation increases the fruit or seed set (Burd, 1994; Knight et al., 2005). Moreover, pollen limitation may have important consequences for plant reproduction and the abundance of plant species (Aizen and Harder, 2007; Fernández et al., 2012). Understanding the causes of pollen limitation in fragmented habitats is thus essential to improve predictions of pollinator reduced in the conservation of plant populations (Eckert et al., 2010; Fernández et al., 2012). Pollen limitation commonly occurs when pollinators deposit incompatible pollen, when pollinators are scarce, or when plants are self-pollinated (Fernández et al., 2012). Most insect-pollinated plants also show evidence of inadequate pollen receipt (García-Camacho and Totland, 2009). In the current study, pollen limitation was more severe in the control group than in the pollen addition group, thereby showing that pollen limitation is an important limiting factor for seed set. In the same habitat, a similar pollen limitation pattern has also been documented in *Ammopiptanthus mongolicus* (Maxim), reaffirming that pollen limitation affects plant reproductive success (Chen et al., 2016).

Pollen limitation is likely to be stronger in small, isolated habitats than in large, well-connected habitats (Aizen and Feinsinger, 1994; Fernández et al., 2012). Pollen limitation may be a consequence of changes in pollinator abundance and may be caused when pollinators are scarce or ineffective (Larson and Barrett, 2000; Ashman et al., 2004; Harder and Aizen, 2010). In a fragmented habitat, the flowers of animal-pollinated species often experience pollen limitation due to unreliable pollinator services (Ashworth et al., 2004). Ksiazek et al. (2012) noted that increases in fragmented habitats affect plant reproductive success due to changes in pollinator visitation rate, density, and diversity. Plant reproduction may be limited by inadequate pollen receipt or resource availability (Griffin and Barrett, 2002; Knight et al., 2005). In *H. ammodendron*, we found that pollinator visitation frequency in the natural habitat was significantly higher than that in the fragmented habitat. However, pollen limitation was similar in the fragmented and natural habitats because pollen transfer relies on wind in both habitats, and wind pollination played an important role in the outcrossing. Therefore, habitat fragmentation affects pollinator visitation frequency and activity but does not reduce seed set.

In previous papers, pollen supplementation experiments have not been very informative because plants may be able to reallocate resources among flowers (Ashman and Schoen, 1997; Ashman, 2004). If added pollen is applied to labeled flowers or inflorescences on a plant, then resources may be reallocated away from untreated flowers to support the seed set of the treated flower. To account for potential resource reallocation, we used a control from the manipulated plants and a procedural control from the non-manipulated ones (Gómez et al., 2010). Our results showed that the seed set of the procedural control flowers (from the non-manipulated plants) are not different from the control flowers (from the manipulated plants), thereby suggesting that resource reallocation did not significantly alter the accompanying flowers, and if resource reallocation existed, it was weak. In fact, in the hypothesis of limited resource allocation, the procedural control would be expected to have a higher rather than a lower seed set than the control.

### Pollinator Activity and Pollination Success of *H. ammodendron* in a Fragmented Habitat

Pollination is essential for the sexual reproduction of seeding plants, and pollinator visitation frequency appears to be a good predictor of pollination success (Tewksbury et al., 2002). In most flowering plants, pollinator visitation, the quality and quantity of pollen and reward systems are major biotic factors influencing the pollination success and seed set (Corbett, 2003). A previous study also showed that a reduction in pollinators causes a decline in the amount of pollen delivered to the stigmas and reduces the probability of cross-pollen transfer, thereby resulting in a reduced seed set (Lennartsson, 2002). Jones and Agrawal (2017) suggest that the relationship between plants and insects is influenced by insects’ behavioral decisions during foraging. Although bumblebees visited more flowers in *Penstemon gentianoides* (Plantaginaceae), the high number of seeds pollinated by hummingbirds highlights the importance of hummingbirds’ foraging strategy (Salas-Arcos et al., 2017). General pollinators experience a high level of environmental variation, and pollinator learning thereby has the potential to have an important role in plant-insect coevolution (Jones and Agrawal, 2017).

The effects of habitat loss and fragmentation could include reduced pollinator diversity and abundance, and the species richness of pollinators also decreases with fragmented habitat isolation (Nayak and Davidar, 2010). Many plant species relying on less effective pollinators may experience serious declines in pollination success if a harsh environment and climate change affect pollinator activity (Michael et al., 2003). Our outcomes also support the view that higher pollinator visitation rates resulted in a higher percentage of seeds. The effects of the arid climate and environment could have reduced plant pollinator diversity and abundance (Gómez et al., 2007). Rodríguez-Cabal et al. (2007) suggested that pollinators are more unreliable and persistently less abundant in arid environments, in part due to habitat fragmentation, which seriously affects pollinator visits. Our results indicated that the number of flowers visited by *A. mellifera* was significantly higher in the natural habitat than in the fragmented habitat because *A. mellifera* preferred to visit areas with greater resource availability.

Chen et al. (2015) noted that the number of species relying solely on insect pollination or wind pollination mechanisms is low, with most species using a combination of both mechanisms. In the present study, the seed set of naturally pollinated flowers in the natural habitat was higher than that in the fragmented habitat, but there were no significant differences between both habitats. The reason for this result is that *H. ammodendron* relies on a combination of wind pollination and insect pollination mechanisms, and wind pollination is critical in the outcrossing system. Krishnana et al. (2012) also suggested that wind pollination conferred an advantage in pollination success when there was a scarcity of pollinators in a fragmented landscape mosaic. Therefore, *H. ammodendron* displayed a highly adaptive pollination system under harsh environmental stress.

## Conclusion

In this study, we found that pollen supplementation affected seed production in the fragmented and natural habitats, and pollen limitation was the important limiting factor for seed set. Our results suggest that higher pollinator visitation rates resulted in a higher percentage of seeds. Moreover, our results also showed that the fragmented habitats reduced the visiting frequency and changed the activity of *A. mellifera*. Understanding the relationships among pollen limitation, pollinator visits, and seed set is critical in designing effective management strategies to increase pollination success in arid environments.

## Author Contributions

MC designed the experiment and wrote the manuscript. X-AZ provided editorial advice.

### Conflict of Interest Statement

The authors declare that the research was conducted in the absence of any commercial or financial relationships that could be construed as a potential conflict of interest.
