# Supplementary material for: Effect of Pollen Limitation and Pollinator Visitation on Pollination Success of Haloxylon ammodendron (C. A. Mey.) Bunge in Fragmented Habitats
Source: Front Plant Sci. 2019 Mar 22;10:327. doi: 10.3389/fpls.2019.00327 (PMC6448003; doi:10.3389/fpls.2019.00327)
Supplement: Supplementary file 1 [file Table_1.DOCX]

**Supplementary material**

**The list of dominant and occasional pollinators in *H. ammodendron*.**

|  | Order | Family | Genus |
| --- | --- | --- | --- |
| *Apis mellifera* | Hymenoptera | Apidae | Apis |
| *Megachile spissula* Cockerell | Hymenoptera | Megachilidae | Megachile |
| *Episyrphus balteatus* | Diptera | Syrphidae | Episyrphus |
| *Pieris rapae* Linne | Lepidoptera | Pieridae | Pieris |
